# Performance of Intrinsic and Modified Graphene for the Adsorption of H_2_S and CH_4_: A DFT Study

**DOI:** 10.3390/nano10020299

**Published:** 2020-02-10

**Authors:** Xin Gao, Qu Zhou, Jingxuan Wang, Lingna Xu, Wen Zeng

**Affiliations:** 1 College of Engineering and Technology, Southwest University, Chongqing 400715, China; g1289547142@163.com (X.G.); wangjx183@163.com (J.W.); Lingnaxu@swu.edu.cn (L.X.); 2 Electrical and Computer Engineering Department, Wayne State University, Detroit, MI 48202, USA; 3 College of Materials Science and Engineering, Chongqing University, Chongqing 400044, China

**Keywords:** H_2_S, CH_4_, adsorption, graphene, first principles

## Abstract

In this study, the adsorption performances of graphene before and after modification to H_2_S and CH_4_ molecules were studied using first principles with the density functional theory (DFT) method. The most stable adsorption configuration, the adsorption energy, the density of states, and the charge transfer are discussed to research the adsorption properties of intrinsic graphene (IG), Ni-doped graphene (Ni–G), vacancy defect graphene (DG), and graphene oxide (G–OH) for H_2_S and CH_4_. The weak adsorption and charge transfer of IG achieved different degrees of promotion by doping the Ni atom, setting a single vacancy defect, and adding oxygen-containing functional groups. It can be found that a single vacancy defect significantly enhances the strength of interaction between graphene and adsorbed molecules. DG peculiarly shows excellent adsorption performance for H_2_S, which is of great significance for the study of a promising sensor for H_2_S gas.

## 1. Introduction

Hydrogen sulfide (H_2_S) is a colorless, toxic, flammable gas which causes pollution to the environment [1,2]. It is the product of various industrial processes, such as natural gas processing and treatment, high-temperature coking, oil refining, and biogas fermentation [3,4]. H_2_S is harmful to human health, as it causes damage to the nervous system, even resulting in coma and death with an increase in concentration [5]. In addition, H_2_S can be oxidized to form SO_2_, which is one of the main sources of acid rain, causing damage to the natural environment and infrastructure [6]. At the same time, H_2_S corrodes equipment during industrial processes and causes serious economic loss [7]. Methane (CH_4_) is a tetrahedral nonpolar molecule, which is colorless, tasteless, and flammable [8]. It is the main component of natural gas, biogas, or pit gas [9]. Relevant research shows that CH_4_ is an important source gas of the greenhouse effect, and the content of methane in the environment is increasing with each passing day [10,11]. In addition, CH_4_ can cause suffocation and even death when the concentration of CH_4_ increases to a certain value [12]. For these reasons, the adsorption of H_2_S and CH_4_ attracts comprehensive research and analysis, as it is very important to design or find a feasible sensor.

Graphene is a two-dimensional hexagonal honeycomb structure formed by a C atom with *sp^2^* hybrid orbitals [13]. It has excellent mechanical, electrical, optical, and thermal conductivity; thus, it is widely used in various fields like gas detection [14,15,16]. Graphene allows easy charge transfer with external gas molecules because of its large specific surface area, high carrier mobility, and good conductivity, which also allows the preparation of high-performance graphene-based sensors [17,18,19]. However, many studies showed that intrinsic graphene has weak physical adsorption of most gas molecules [20,21]. Therefore, in order to solve this problem, the concepts of doped graphene [22] and vacancy defect graphene [23] were proposed. Many studies showed that the introduction of defects or doped atoms in graphene can effectively strengthen the charge transfer between graphene and gas molecules, improve the adsorption capacity of certain gas molecules, and enhance the sensitivity and selectivity of graphene-based sensors [24,25]. In addition, graphene oxide can be obtained by adding different kinds of oxygen-containing functional groups on the surface of graphene, such as hydrocarbon, carboxyl, epoxy, carbonyl, etc. [26,27]. The presence of oxygen-containing groups not only makes graphene oxide have chemical stability, but also provides the active site of surface modification and a large specific surface area [28]. Therefore, graphene oxide may be an ideal and promising sensor material.

Ganji et al. analyzed the adsorption process of H_2_S on the surface of graphene and Pt-doped graphene, and they found that the latter had higher charge transfer and lower adsorption energy [29]. Wang et al. confirmed that graphene modified by Cu nanoparticles showed good glucose detection performance, with a low detection limit, high sensitivity, fast response time, and reliable stability [30]. Ovsianytskyi et al. found that graphene decorated with Ag nanoparticles had high selectivity and sensitivity to H_2_S gas, because Ag doping improved the conductivity of graphene and enhanced the charge transfer between graphene and H_2_S [31]. Gui et al. studied the gas sensing of intrinsic graphene and graphene oxide for ClO_2_ and its decomposed species, and they found that graphene oxide was better than intrinsic graphene, showing an excellent adsorption of ClO_2_ [32]. Liu et al. studied the adsorption by graphene oxide of water molecules, and they found that it had good water adsorption [33].

In this study, Ni was selected to form Ni-doped graphene. As a cheap and promising metal catalyst, Ni attracted more and more attention in recent years, and it was widely used in many kinds of materials to achieve better results [34,35]. However, at present, there is no comparison of the gas adsorption capacity of intrinsic graphene (IG), vacancy defect graphene (DG), Ni-doped graphene (Ni–G), and graphene oxide (G–OH). Therefore, H_2_S and CH_4_ were selected as the adsorption object, and the adsorption capacity of four graphene models toward H_2_S and CH_4_ was compared by analyzing differences in adsorption energy, charge transfer, appearance change, density of state (DOS), partial density of state (PDOS), and electron density in this paper. These studies may provide some theoretical basis for the search of sensor materials with high sensitivity to H_2_S or CH_4_ gas, and they may also provide direction for the research of modified graphene-based sensors.

## 2. Theory and Simulation

All calculations in the paper were carried out using the Dmol3 module of Material Studio (MS) [36,37]. The adsorption process was studied using first principles with the density functional theory (DFT) method [38]. The Perdew–Burke–Ernzerhof (PBE) functional was chosen to modify the parameterized generalized gradient approximation (GGA) to obtain the correct ground-state structure, and the exchange correlation energy under all relax conditions was processed [39]. In order to ensure good accuracy, convergence tolerances for geometry optimization including quality, energy, maximum Force, and maximum displacement were set to customized, 1 × 10^−6^ Ha, 0.002 Ha/Å, and 0.005 Å, respectively. Spin unrestricted was chosen to neglect the effect of spin polarization. The double numerical plus polarization (DNP) was selected, and the DFT semi-core pseudopotential (DSPP) was applied for core treatment because of its relativistic effects. The k point of the Brillouin zone integration and the self-consistent (SCF) field tolerance were set to 6 × 6 × 1 and 1 × 10^−5^ Ha in this study. The direct inversion of iterative subspace (DIIS) was set to 6 to accelerate the convergence of SCF charge density.

In this paper, the adsorption energy (*E_ads_*) of gas molecules adsorbed on four kinds of graphene-based materials was calculated using the following equation:

$$E_{ads}=E_{\left(gas-substrate\right)}-E_{\left(substrate\right)}-E_{\left(gas\right)},$$

where *E_(gas-substrate)_*, *E_(substrate)_*, and *E_(gas)_* are the total energies of the graphene system after gas molecule adsorption, the graphene system before gas molecule adsorption, and a single gas molecule, respectively [40,41].

In addition, for a simpler and clearer representation of all modeled structures, unique alphanumeric designations were assigned, as shown in Table 1. The corresponding designations are used in this paper.

## 3. Results and Discussion

### 3.1. Establishment and Analysis of All the Models

**Figure 1:**
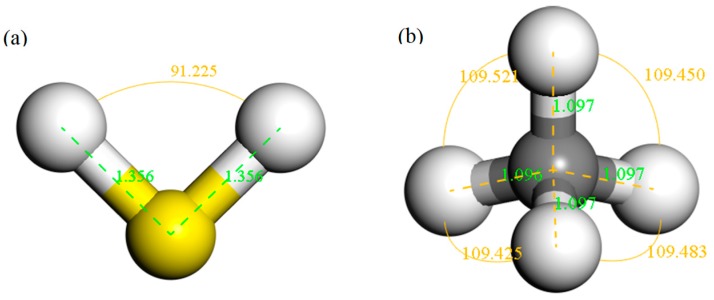
The structures of H_2_S (**a**) and CH_4_ (**b**) molecules.

**Figure 2:**
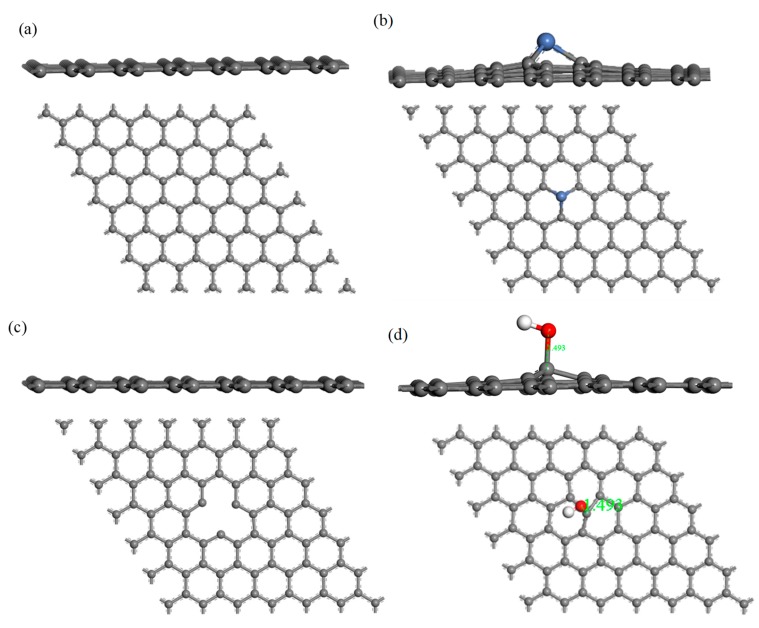
The side and top views of IG (**a**), Ni–G (**b**), DG (**c**), and G–OH (**d**) structures.

**Figure 3:**
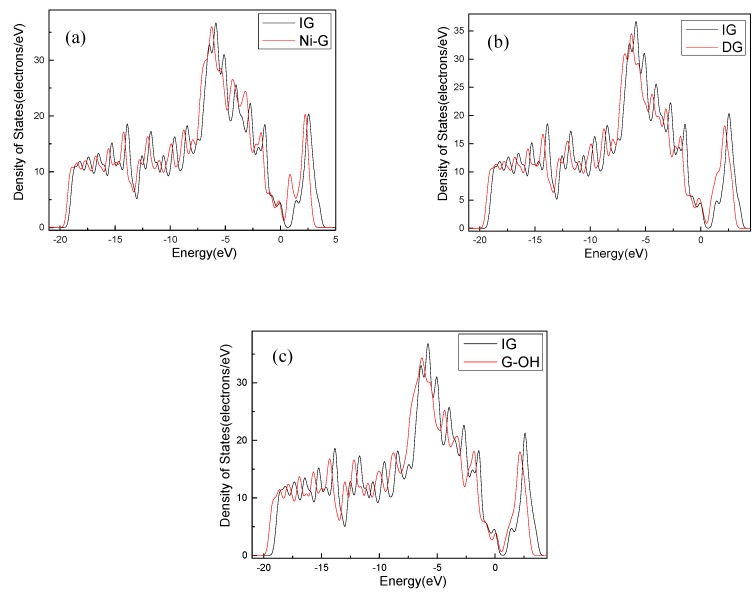
The DOS comparison of Ni–G (**a**), DG (**b**), and G–OH (**c**) with IG.

The structure of H_2_S and CH_4_ molecules was established, and the optimized structures are shown in Figure 1. The S–H bond length and the bond angle of the H_2_S molecule were 1.356 Å and 91.225°, respectively. The C–H bond length and the bond angle of the CH_4_ molecule were about 1.097 Å and 109.500°, respectively. These values are consistent with the literature [42,43]. IG was constructed using a supercell of 6 × 6 (72 atoms) with a vacuum distance of 20 Å and a slab position of −10 Å to avoid interactions between adjacent layers and the establishment of monolayer graphene. Ni-G was built though substituting the C atom numbered 43 (C_43_) with an Ni atom. DG was obtained by removing C_43_. G–OH was formed by adding a hydrocarbon functional group to C_43_. The four optimized graphene models are shown in Figure 2, and the structural parameters are listed in Table 2. The comparison of the DOS of the three graphene systems with that of the intrinsic graphene is shown in Figure 3.

The C–C bond length and the bond angle of IG after optimization were 1.420 Å and 120.020°, respectively. The values are consistent with the theoretical values in the literature, which indicates that the IG model can be used for further calculations [44]. There is no charge transfer between C atoms in intrinsic graphene, and the gain and loss of all C atoms are zero. The structure of the optimized Ni-doped graphene model changed obviously. The Ni atom protruded upward and formed a local three-dimensional structure with the surrounding C atoms, due to the change in stress caused by doping of Ni atom. Compared with intrinsic graphene, the bond length of Ni–C was longer than that of C–C. The charge transfer between the graphene sheet and Ni atom was 0.013 e, which indicates that graphene lost electrons and the Ni atom obtained electrons. It can be seen from the Figure 3a that, because of the doping of the Ni atom, the Fermi level entered into the valence band, which resulted in the system showing obvious metal characteristics. It can be seen from Table 1 that the bond length and the bond angle of the optimized vacancy defect graphene changed little. However, a charge transfer of 0.005 e and a spin magnetic moment of 2.002 existed, which indicated that the DG system showed magnetism. According to Figure 3b, the Fermi level slightly moved into the valence band, leading to the enhancement of the conductivity. It can be seen from Table 1 and Figure 2d that the adjacent carbon six-membered rings on the graphene sheet underwent severe deformation, and due to the hydrocarbon functional group, the C atoms protruded upward to form the local *sp^3^* configuration. The charge transfer between the graphene monolayer and hydrocarbon group was 0.233 e. The former lost electrons while the latter gained electrons. However, the graphene oxide system was non-magnetic. Similarly, the Fermi level moved into the valence band, but the strength was between that of the DG and Ni–G systems, as shown in Figure 3c.

### 3.2. IG, Ni–G, DG, and GO Adsorption of H_2_S

**Figure 4:**
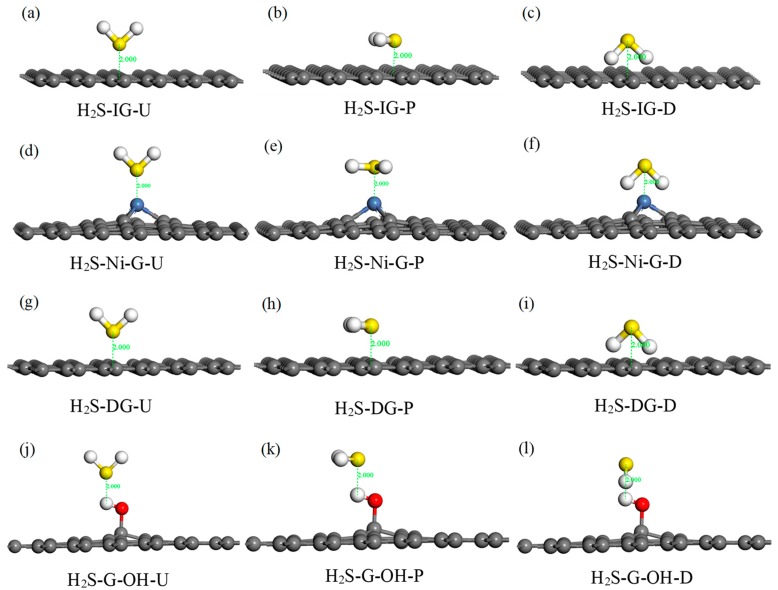
Different adsorption configurations of H_2_S adsorbed on IG (**a**–**c**), Ni–G (**d**–**f**), DG (**g**–**i**), and G–OH (**j**–**l**).

H_2_S is a kind of polar molecule; thus, three typical adsorption configurations were selected in this study: (1) the H_2_S molecule was perpendicular to IG, Ni–G, DG, or G–OH, and the S atom was close to the C, Ni, C, or H atom (abbreviated as H_2_S–IG–U, H_2_S–Ni–G–U, H_2_S–DG–U, H_2_S–G–OH–U); (2) the H_2_S molecule was parallel to IG, Ni–G, DG, or G–OH, and the S atom was above the C, Ni, C, or H atom (abbreviated as H_2_S–IG–P, H_2_S–Ni–G–P, H_2_S–DG–P, H_2_S–G–OH–P); (3) the H_2_S molecule was perpendicular to IG, Ni–G, DG, or G–OH, and the S atom was far away from to the C, Ni, C, or H atom (abbreviated as H_2_S–IG–D, H_2_S–Ni–G–D, H_2_S–DG–D, H_2_S–G–OH–D). Initial adsorption distance was set to 2 Å. The 12 initial configurations of H_2_S molecule adsorbed on IG, Ni–G, DG, and G–OH are shown in Figure 4, and the adsorption energy of these configurations is listed in Table 3. According to the adsorption energy in Table 3, it can be seen that different initial adsorption configurations affected the adsorption between H_2_S and the four graphene systems; thus, four adsorption models (H_2_S–IG–U, H_2_S–Ni–G–P, H_2_S–DG–D, H_2_S–G–OH–P) were chosen for further study. In order to simply describe the four systems, we use H_2_S–IG, H_2_S–Ni–G, H_2_S–DG, and H_2_S–G–OH instead.

**Figure 5:**
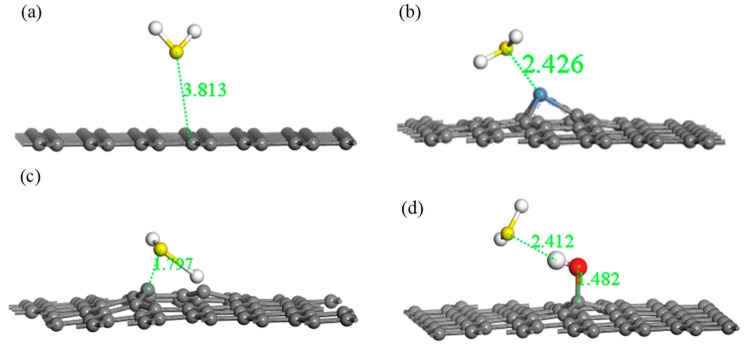
Optimized models of H_2_S adsorbed on IG (**a**), Ni–G (**b**), DG (**c**), and G–OH (**d**).

Adsorption energy, the final adsorption distance, and charge transfer are three important aspects for describing the strength of adsorption between gas molecule and the graphene system. A lower adsorption energy results in a shorter final adsorption distance, a larger charge transfer amount, and a stronger adsorption effect. Figure 5 shows the four optimized adsorption models with respect to H_2_S, and Table 4 lists the relevant parameters of these models.

From Table 4, the value of the final adsorption distance was 3.811 Å in the system of IG adsorption of H_2_S, which was further than the initial distance. Furthermore, the charge transfer was 0.004 e, which is very poor. Moreover, the absolute value of the adsorption energy was extremely small. The lengthy final adsorption distance, the poor charge transfer, and the low absolute value of the adsorption energy indicates that intrinsic graphene had a weak adsorption capacity for H_2_S. Compared with intrinsic graphene, the adsorption distance reduced from 3.811 Å to 2.426 Å, showing a stronger interaction between Ni–G and H_2_S. In addition, the absolute value of adsorption energy was significantly increased, but Ni–G did not form a strong chemical adsorption for H_2_S. A strong charge transfer existed between Ni–G and the H_2_S molecule, resulting in a value of 0.233 e, which illustrates that the doping of Ni significantly improved the electron transfer ability. Meanwhile, it can be seen that graphene obtained electrons and the H_2_S molecule lost electrons during the adsorption process. Therefore, Ni doping could slightly improve the adsorption ability of graphene with respect to H_2_S. Analogously, for the system of G–OH adsorption of H_2_S, the absolute value of adsorption energy increased evidently, which showed a strong chemical adsorption. The adsorption distance was 2.412 Å, which was smaller than that of IG and Ni–G systems. The charge transfer was 0.054 e, which illustrates that the hydrocarbon functional group acted as the bridge of charge transfer between graphene and H_2_S. However, the increase was far less than that of Ni–G. The absolute value of adsorption energy was 2.934 eV in the system of G–OH adsorbed to H_2_S, which was significantly increased compared with the other three systems. The adsorption distance decreased, and the charge transfer was 0.172 e. It can be seen that the increasing range of charge transfer was smaller than that of adsorption energy and adsorption distance, which was probably due to the electron transfer in DG interfering with the electron transfer between H_2_S and DG. The extremely low adsorption energy, the short adsorption distance, and the good charge transfer amount showed the extremely strong chemical adsorption between DG and the H_2_S molecule, which shows that graphene is an excellent material for H_2_S adsorption. In addition, Ni–G and G–OH can also be viable options.

**Figure 6:**
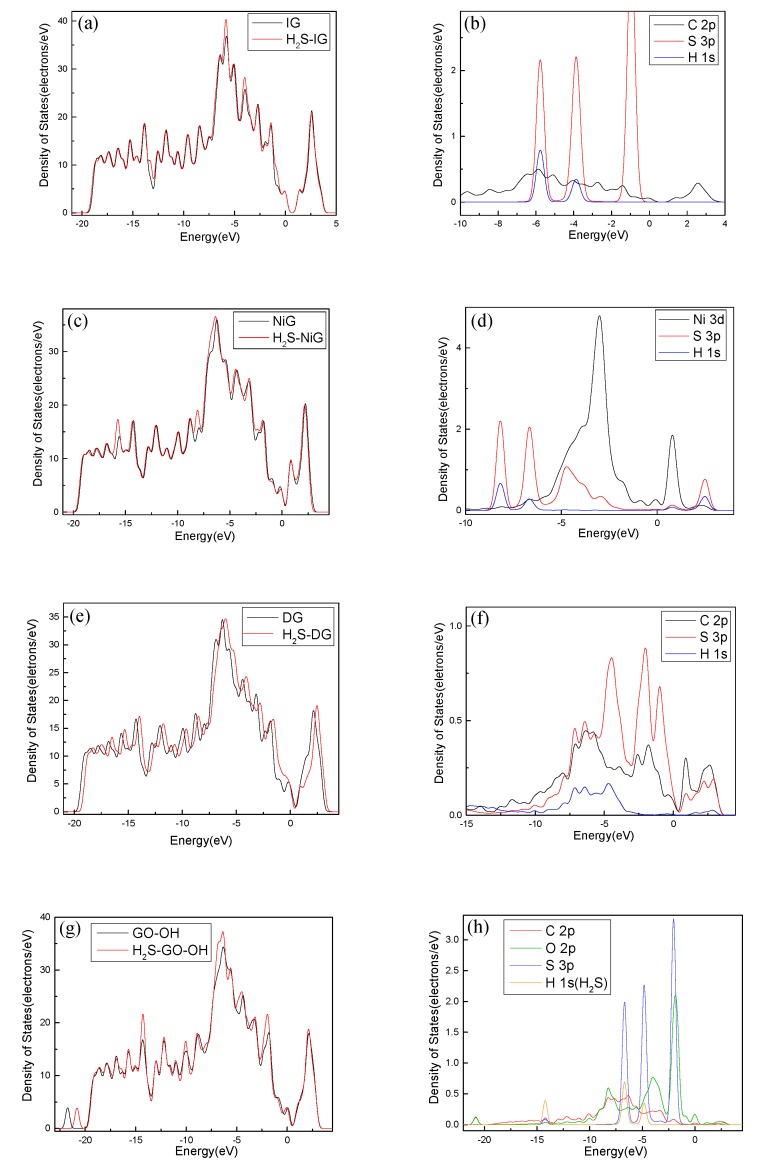
The DOS and PDOS of H_2_S adsorbed on IG (**a**, **b**), Ni–G (**c**, **d**), DG (**e**, **f**), and G–OH (**g**, **h**).

**Figure 7:**
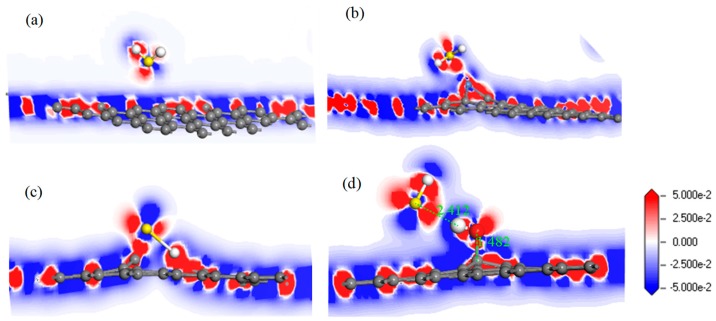
The electron density difference of H_2_S adsorbed on IG (**a**), Ni–G (**b**), DG (**c**), and G–OH (**d**).

For further study of the adsorption between the four graphene systems and the H_2_S molecule, the DOS and the PDOS are shown in Figure 6, and the electron density difference of the four systems is shown in Figure 7, where a change from blue to red indicates a gradual increase in charge density. The DOS before and after the adsorption of H_2_S on IG changed little from Figure 6a. The DOS coincided at the Fermi level, and only slightly increased at −4 eV and −6 eV. We analyzed the PDOS distribution of the outermost orbitals of the main characteristic atoms. It can be found from Figure 6b that the C *2p* orbital had almost no overlapping peak with H *1s* and S *3p* orbitals. In addition, there was no effective contact of charge density between IG and H_2_S molecule from Figure 7a. Therefore, it can be further confirmed that the intrinsic graphene showed only weak physical adsorption of H_2_S. There was almost no change in DOS at the Fermi level after the adsorption of H_2_S by Ni–G, but the overall DOS slightly shifted to the left from Figure 6c. This shows that the charge transfer was easier. According to Figure 6d, the PDOS of the Ni *3d*, H *1s*, and S *3p* orbitals was analyzed. The Ni *3d*, H *1s*, and S *3p* orbitals had overlapped peaks surrounding −8 eV, −4 eV, −12 eV, 1 eV, and 2.5 eV. Fewer overlapping areas result in insignificant orbital effects. Moreover, the charge density of Ni–G was similar to that of the H_2_S molecule from Figure 7b. Therefore, the charge transfer between Ni–G and H_2_S was relatively strong, showing quite strong polarization. The whole curve of DOS moved to the right, and the DOS at the Fermi level showed a little decline from Figure 6e. It can be found from Figure 6f that the hybrid effect between C *2p*, H *1s*, and S *3p* orbitals was obvious, as reflected in the overlapped peaks from −7.5 eV to 3 eV. Figure 7c shows the strong charge transfer, indicating great polarization. The DOS from Figure 6g of G–OH adsorbing H_2_S showed varying degrees of increase surrounding −14 eV, −8 eV, −6.5 eV, −4.8 eV, and −2 eV. According to the PDOS from Figure 6h, the valence band was mainly composed of C *2p*, S *3p*, O *2p*, and H *1s* orbitals. There were obvious overlapping peaks near −14 eV, −8 eV, −6.5 eV, −4.8 eV, and −2 eV, which indicates the strong hybridization and bonding between the orbitals. Meanwhile, there was no effective contact of charge density between G–OH and the H_2_S molecule from Figure 7d; thus, the polarization was very weak, which is consistent with the small amount of charge transfer. In general, the adsorption system of DG with respect to the H_2_S molecule showed an excellent orbital effect and a strong polarization effect. Therefore, it is obvious that DG graphene exhibited the best adsorption performance for H_2_S molecules. The strong chemisorption, charge transfer, and orbital hybridization suggest that the DG-based sensor may be used to detect H_2_S gas.

### 3.3. IG, Ni–G, DG, and GO Adsorption of CH_4_

**Figure 8:**
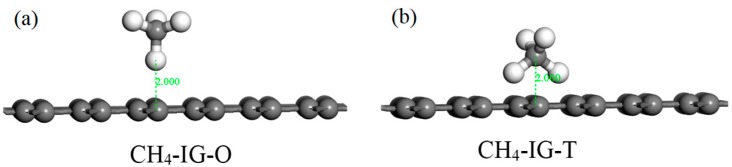
The initial configurations (CH_4_–IG–O (**a**) and CH_4_–IG–T (**b**)) of CH_4_ adsorbing on IG.

According to the structure of the CH_4_ molecule, two initial adsorption configurations were designed. As shown in Figure 8, we take intrinsic graphene as an example. The C atom faced the graphene plane (abbreviated as CH_4_–IG–O) in Figure 8a, and two C atoms symmetrically faced the graphene plane (abbreviated as CH_4_–IG–T) in Figure 8b. Similarly, the other three graphene adsorption models used the same two initial configurations. Initial adsorption distance was set to 2 Å. The adsorption energy of the eight initial configurations are listed in Table 5, and four adsorption models (CH_4_–IG–T, CH_4_–Ni–G–T, CH_4_–DG–O, CH_4_–G–OH–T) were chosen for the further study. Similarly, CH_4_–IG, CH_4_–Ni–G, CH_4_–DG, and CH_4_–G–OH were the terms used henceforth. It can be seen that different adsorption configurations had little effect on the adsorption of CH_4_, which may have been caused by the non-polarity of the CH_4_ molecule.

**Figure 9:**
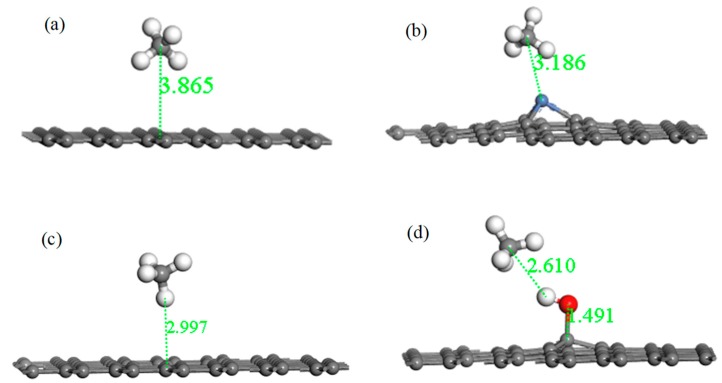
The optimized configurations of CH_4_ adsorbed on IG (**a**), Ni–G (**b**), DG (**c**), and G–OH (**d**).

Figure 9 shows the adsorption structures of the four systems after optimization, and parameters such as absorption distance, bond length, bond angle, and charge transfer are listed in Table 6. For the system of IG adsorbing the CH_4_ molecule, it can be found that the intuitive structures of both IG and CH_4_ remained substantially unchanged. The adsorption distance after optimization was 3.865 Å, which was much greater than the initial distance. The adsorption energy was −0.022 eV, and the charge transfer was −0.002 e. The long distance, the bad adsorption energy, and little charge transfer indicate that the adsorption capacity of intrinsic graphene for CH_4_ was extremely poor. After Ni–G adsorbing CH_4_, the Ni–C bond length of Ni–G increased, but the form of CH_4_ was basically unchanged. The distance was 3.186 Å, which was shortened. The value of adsorption energy and charge transfer increased. This shows that the adsorption of this system was much stronger, and the doping of Ni improved the adsorption capacity of IG. The charge transfer between Ni–G and CH_4_ was negative, which shows that the Ni–G system lost electrons and the CH_4_ molecule gained electrons. The value of adsorption energy greatly increased, and the distance effectively shortened. The defect of graphene brought about excellent adsorption effects. However, due to the influence of electron transfer in the defective graphene system, the value of charge transfer was 0.004 e. Finally, for the system of G–OH adsorbing CH_4_, the adsorption distance was greatly reduced, which allowed improving the adsorption effect. The small adsorption energy and weak charge transfer showed that the effect of adsorption of CH_4_ by G–OH was not good. Speaking generally, the adsorption capacity of the three graphene systems was enhanced compared with that of intrinsic graphene. Among them, the adsorption performance of DG was the strongest, followed by Ni–G.

**Figure 10:**
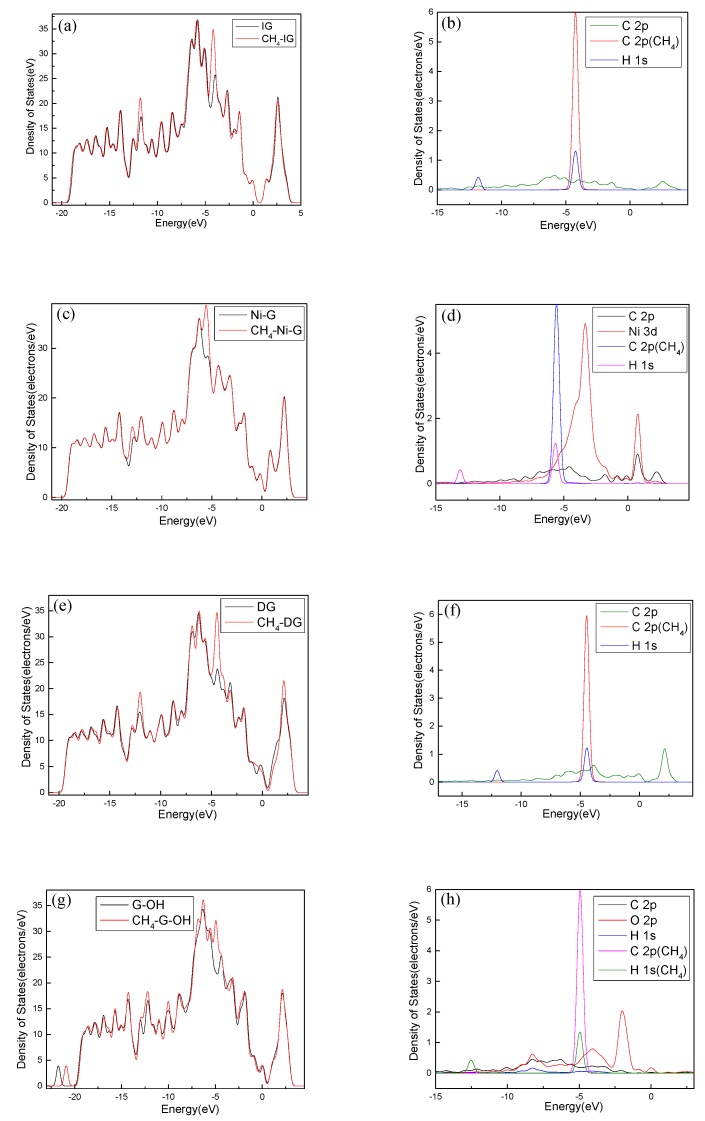
The DOS and PDOS of CH_4_ adsorbed on IG (**a**,**b**), Ni–G (**c**,**d**), DG (**e**,**f**), and G–OH (**g**,**h**).

**Figure 11:**
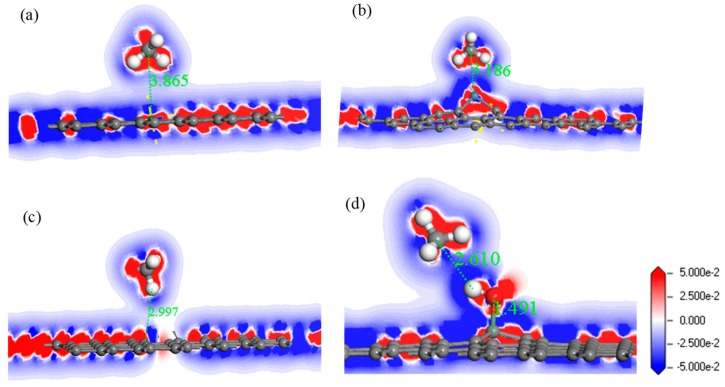
The electron density difference of CH_4_ adsorbed on IG (**a**), Ni–G (**b**), DG (**c**), and G–OH (**d**).

In order to analyze the adsorption performance of the systems, the DOS, PDOS, and the electron density difference are discussed. Figure 10 shows the DOS and PDOS of the CH_4_ molecule adsorbed on IG, Ni–G, DG, and G–OH, and the electron density difference of the four adsorption systems are shown in Figure 11. It can be found in Figure 10a that the DOS of IG before and after the adsorption of CH_4_ showed almost no difference, but there was a significant increase at −12 eV and −4 eV. By comparing Figure 10b, it can be seen that the PDOS at −4 eV was composed of C *2p*, C *2p* (the C atom of CH_4_), and H *1s* orbitals, and the PDOS at −12 eV was composed of C *2p* and H *1s* orbitals. The C *2p* (CH_4_) and H *1s* orbitals had overlapped peaks surrounding −4 eV, and a small peak of the H *1s* orbital was at −14 eV, which was the reason for the increase in DOS at these two points. As shown in Figure 10c, the DOS increased surrounding −13 eV and −5.5 eV, which was composed of H *1s*, C *2p* (CH_4_), and C *2p* (Ni–G). From Figure 10d, the overlapped peaks appeared around −5.5 eV and 0.5 eV, reflecting the hybridization effect. In the system of DG adsorbing CH_4_, the DOS changed at the Fermi level and increased substantially at −12 eV and −4.5 eV, as shown in Figure 10e. PDOS mainly consisted of C *2p*, C *2p* (the C atom of CH_4_), and H *1s* orbitals. It can be found from Figure 10f that overlapping peaks were around −4.5 eV. As shown in Figure 10g, the DOS at −5 eV had an evident increase. Combined with the PDOS shown in Figure 10h, it can be seen that the PDOS was composed of C *2p*, O *2p*, H *1s*, C *2p* (the C atom of CH_4_), and H *1s* (the H atom of CH_4_) orbitals, and there were overlapping peaks from −10 eV and −2.5 eV, showing strong hybridization. It can be seen from Figure 11 that there was no direct and effective contact between the CH_4_ molecule and the four graphene systems; thus, the charge transfer of the four adsorption systems was not strong. In general, the three kinds of graphene shortened the adsorption distance, and they strengthened the adsorption energy and charge transfer. Among them, DG showed the best adsorption performance to CH_4_. However, the three modified graphene specimens did not produce strong chemical adsorption for CH_4_; thus, we think that they may be not suitable for CH_4_ gas detection.

Above, we analyzed the adsorption systems of three kinds of modified graphene for H_2_S and CH_4_. It can be clearly seen that different modification methods can improve the adsorption capacity of intrinsic graphene to different degrees. However, compared with CH_4_, the three modified graphene specimens had better adsorption capacity for H_2_S, with DG particularly showing excellent adsorption performance for H_2_S, beyond that of IG, Ni–G, and G–OH. Therefore, we carried out a comparative analysis of the research on the adsorption of H_2_S by Ni–G, DG, and G–OH with other published research work, as shown in Table 7. These values were obtained using different DFT functions. GO is the abbreviation of graphene oxide. According to these data, we found that DG has an excellent adsorption effect on H_2_S, which is reflected in its stronger adsorption energy, short adsorption distance, and large charge transfer value. Therefore, DG is a promising material for the detection of H_2_S, which can be used in further experimental research.

## 4. Conclusions

The adsorption of H_2_S and CH_4_ molecules on intrinsic graphene, Ni–doped graphene, single vacancy defect graphene, and graphene oxide was investigated using first principles to study the adsorption performance of functionalized graphene with respect to gas molecules. The adsorption energy, density of states, charge transfer, and electron density difference were discussed in terms of their effect on the adsorption of H_2_S and CH_4_ in different orientations. The results revealed that H_2_S and CH_4_ both show weak physical adsorption on intrinsic graphene. The three kinds of modified graphene improved the adsorption capacity of intrinsic graphene to varying degrees. Among them, DG showed the best adsorption performance for H_2_S and CH_4_. However, compared with the adsorption of CH_4_, DG had excellent adsorption capacity for the H_2_S molecule, with an adsorption energy value of −2.934 eV, an adsorption distance of 1.797 Å, a transfer charge of 0.172 e, and strong orbital hybridization in PDOS. Therefore, DG is a promising material for H_2_S detection which can be used for further experimental research.

## Figures and Tables

**Table 1:** The unique alphanumeric designations of all modeled structures.

Model	Designation	Model	Designation	Model	Designation
Intrinsic graphene	IG	H_2_S adsorbed on IG	H_2_S–IG	CH_4_ adsorbed on IG	CH_4_–IG
Ni-doped graphene	Ni–G	H_2_S adsorbed on Ni–G	H_2_S–Ni–G	CH_4_ adsorbed on Ni–G	CH_4_–Ni–G
Vacancy defect graphene	DG	H_2_S adsorbed on DG	H_2_S–DG	CH_4_ adsorbed on DG	CH_4_–DG
Graphene oxide	G–OH	H_2_S adsorbed on G–OH	H_2_S-G–OH	CH_4_ adsorbed on G–OH	CH_4_-G–OH

**Table 2:** The structural parameters of IG, Ni–G, DG, and G–OH.

	IG	Ni–G	DG	G–OH
Bond length (Å)	(C–C) 1.420, 1.420, 1.420	(Ni–C) 1.796, 1.799, 1.799	(C–C) 1.399, 1.368	(C–O) 1.493 (C–C) 1.648, 1.555, 1.364
Bond angle (°)	(C–C–C) 120.020	(C–Ni–C) 94.287	(C–C–C) 123.558	(C–C–C) 79.203, 115.470, 144.921
Charge transfer (e)	0	−0.013	−0.005	−0.233
Spin (μ_B_)	0	0	−2.002	0

**Table 3:** The E_ads_ of H_2_S adsorbed on IG, Ni–G, DG, and G–OH.

Models	E_ads_ (eV)			
	H_2_S–IG	H_2_S–Ni–G	H_2_S–DG	H_2_S–G–OH
U	−0.038	−0.698	−1.173	−1.256
P	−0.019	−0.699	−1.149	−1.263
D	−0.025	−0.684	−2.934	−1.258

**Table 4:** The parameters of the H_2_S adsorbed on IG, Ni–G, DG, and G–OH.

Different System	Absorption Energy (eV)	Absorption Distance (Å)	Bond Length (Å)		Bond Angle (°)	Charge Transfer (e)
H_2_S–IG	−0.038	3.811	S–H_1_ (1.355) S–H_2_ (1.355)	C–C 1.423	90.707	+0.004
H_2_S–Ni–G	−0.699	2.426	S–H_1_ (1.359) S–H_2_ (1.358)	Ni–C 1.844, 1.848, 1.809	92.174	+0.233
H_2_S–DG	−2.934	1.797	S–H_1_ (1.358) S–H_2_ (2.146)	C–C 1.452, 1.435	86.093	+0.172
H_2_S–G–OH	−1.263	2.412	S–H_1_ (1.356) S–H_2_ (1.356)	C–O 1.482 C–C 1.501	91.456	+0.054

**Table 5:** The adsorption energy of CH_4_ adsorbing on IG, Ni–G, DG, and G–OH.

Models	E_ads_ (eV)			
	CH_4_–IG	CH_4_– Ni–G	CH_4_–DG	CH_4_–G–OH
O	−0.017	−0.095	−0.154	−0.040
T	−0.022	−0.099	−0.153	−0.047

**Table 6:** The parameters of the CH_4_ adsorbed on IG, Ni–G, DG, and G–OH.

Different System	Absorption Energy (eV)	Absorption Distance (Å)	Bond Length (Å)		Bond Angle (°)	Charge Transfer (e)
CH_4_–IG	−0.022	3.865	C–H (1.096)	C–C 1.421	109.449	−0.002
CH_4_–Ni–G	−0.099	3.186	C–H (1.100)	Ni–C 1.808, 1.801, 1.798	111.341	−0.041
CH_4_–DG	−0.154	2.997	C–H (1.097)	C–C 1.399, 1.474	109.466	−0.004
CH_4_–G–OH	−0.047	2.610	C–H (1.097)	C–O 1.491 C–C 1.500	109.982	−0.009

**Table 7:** The comparison of simulation results of different graphene-based materials for different gas molecules. GO-graphene oxide.

Gas	Material	E_ads_ (eV)	Adsorption Distance (Å)	Charge Transfer (e)	Reference
CO	DG	−1.864	1.329	0.24	[25]
Cl_2_	Ni–G	−0.633	2.742	0.051	[32]
H_2_O	GO	−0.72	/	0.039	[45]
H_2_O	Y–GO	−1.38	/	0.044	[45]
CO_2_	Ni–G	−0.85	3.4	0.15	[46]
H_2_S	Pt–G	−2.034	2.274	0.035	[47]
H_2_S	Pd–G	−1.228	2.202	0.113	[47]
H_2_S	Ni–G	−0.699	2.426	0.233	This work
H_2_S	DG	−2.934	1.797	0.172	This work
H_2_S	G–OH	−1.263	2.412	0.054	This work
